# Effects of P38 MAPK Pathway Inhibition on the Metabolism of Periodontal Ligament Fibroblasts During Inflammation

**DOI:** 10.1111/jcmm.71223

**Published:** 2026-06-01

**Authors:** Cheng Qian, Yifan Gu, Feiyan Gao, Zenan Xu, Jiahui Li, Shuyu Liu, Rui He, Liangjun Zhong, Jintao Wang

**Affiliations:** ^1^ Department of Stomatology Shaoxing Central Hospital Zhejiang China; ^2^ Department of Stomatology Shaoxing KEQIAO Women&Children's Hospital Zhejiang China; ^3^ School of Stomatology Hangzhou Normal University Zhejiang Hangzhou China; ^4^ Department of Stomatology Affiliated Hospital of Hangzhou Normal University Zhejiang Hangzhou China

**Keywords:** Matrix metalloproteinases, metabolism, P. gingivalis, p38 MAPK pathway, periodontal inflammation

## Abstract

During the development of periodontitis, osteoclast function is activated by the MAPK pathway. MMPs are able to participate in cross‐activation and self‐activation cascades, thereby modulating gene expression for osteoclast differentiation. Among the MAPK family, the p38 family has a particularly significant impact on the development of chronic inflammation in periodontal tissue. Activation of p38 MAPK signalling directly or indirectly mediates the expression of inflammatory cytokines, thereby synergistically stimulating MMP production. This experiment aims to understand how p38 MAPK affects inflammatory hPDLFs treated with Pseudomonas gingivalis. Compared with the control group, secretion of MMP‐2, −1, and −3 of hPDLFs treated with *P. gingivalis* ‐LPS was significantly increased and was closely related to the concentration of *P. gingivalis* ‐LPS. Cell scratching and CCK‐8 experiments revealed that MMP‐1, −2, and −3 inhibited cell proliferation and motility. To explore the signalling pathways involved in p38 MAPK regulation, hPDLFs were treated with *P. gingivalis* ‐LPS alone or in combination with a p38 MAPK kinase inhibitor. The results suggest that MMP‐1, −2, and −3 can serve as salivary biomarkers for the chronic inflammatory disease periodontitis and regulate inflammation through the p38 MAP kinase pathway.

**Abbreviations**
(CCK‐8): Cell Counting Kit‐8
(DMEM): Dulbecco's modified Eagle medium
(ELISA): Enzyme‐Linked Immunosorbent Assay
(hPDLFs): human periodontal ligament fibroblasts
(JNK): c‐Jun N‐terminal kinase
(LPS): lipopolysaccharide
(MAPK): The mitogen‐activated protein kinase
(MMPs): Matrix metalloproteinases
(p38 MAPK): the p38 mitogen‐activated protein kinase
(PDLFs): Periodontal ligament fibroblasts
(*P.gingivalis*):
*Porphyromonas gingivalis*

(RT‐PCR): Reverse transcription polymerase chain reaction
(SB239063): p38 inhibitors

## 1 Introduction

Chronic periodontitis is an inflammatory disease caused by the dental plaque biofilm and generally leads to bleeding and oral odour [1]. Ultimately causes tooth loss [2]. Periodontal ligament fibroblasts (PDLFs) undergo significant changes in the development of periodontitis.

The study explored the role of *Porphyromonas gingivalis* ( *P. gingivalis* ) in the progression of periodontitis, causing the destruction of the periodontal ligament when immune balance is disrupted [3]. In addition, lipopolysaccharide (LPS) is a virulence factor of *P. gingivalis, and* it plays a very influential role in periodontitis.

Some research has elucidated the relevance between the pathogenesis of inflammatory and metabolic changes [4]. Studies show that altered cellular metabolic patterns can directly lead to inflammation. Mitogen‐activated protein kinase (MAPK) pathway plays a very influential role in periodontitis, influencing cellular responses, e.g., cytokine secretion, migration and metalloproteinase secretion [5]. MAPK pathway includes 3 kinase families: c‐Jun N‐terminal kinase (JNK), extracellular signal‐regulated kinase (ERK) and p38 mitogen‐activated protein kinase (p38 MAPK), these present highly expressed in the gingival fluid [6]. Together, above all, kinases regulate proliferative metabolism, cell death, and inflammatory manifestations. There is growing proof that modulating kinases has the potential to treat periodontitis. Therefore, targeting the MAPK pathway has become a hopeful treatment [7]. It is worth noting that the p38 MAPK is an important member of the MAPK signalling pathway [8]. Research suggests that p38 MAPK plays a key role in regulating cell survival [9].

One of the very influential factors in the ECM homeostasis is matrix metalloproteinase (MMP). MMP is to a great extent expressed in various organs and tissues of our body. MMP is produced by many human cells, including fibroblasts. MMPs break down the ECM through many types and targets. It is well known that MMP‐9 and MMP‐8 are the richest MMPs in the periodontal tissue and are currently among the most promising and well‐established biomarkers for oral periodontitis [10]. But periodontal ligament fibroblasts can also produce MMP‐1, 2, 3, 7, and 11, while MMP‐1, −2, and −3 are currently less studied. The most important MMP‐1 s that play a role in degradation are those that degrade type 1 and type 3 collagen. MMP‐2 is a gelatinase enzyme responsible for breaking down collagen in the basement membrane. MMP‐3, as the matrix lysine, can not only affect the ECM but also activate the pro‐MMP‐1 [10].

Our study aimed to explore the influence of the p38 MAPK in the human periodontal ligament fibroblasts (hPDLFs) dealt with the *Porphyromonas gingivalis* lipopolysaccharide ( *P. gingivalis* ‐LPS). We aim to discover the role of p38 MAPK in the matrix metalloproteinase (MMP) metabolism of periodontitis.

We obtained expression results of MMP‐2, −1, and −3 in the hPDLFs and determined MMPs generated in response to *P. gingivalis* ‐LPS treatment. Then, we studied the mechanisms of molecular. Our study aimed to explore how the MAPK pathway in the hPDLFs causes periodontitis by *P. gingivalis* ‐LPS.

## 2 Methods

### 2.1 Cells Culture

We used Dulbecco's modified Eagle medium (DMEM) from Gibco, USA, to culture hPDLFs (Procell, CP‐H136). The hPDLFs were put in the humidified incubator, which was controlled at 37°C, 95% air and 5% carbon dioxide. Then we trypsinised the hPDLFs with the Trypsin/EDTA (Sigma, USA). Additionally, hPDLFs were handled without or with p38 inhibitors (SB239063). And after one hour of preprocessing, the hPDLFs were exposed to 100 μg/mL of the *P. gingivalis* ‐LPS (Ultrapure; InvivoGen) to cause inflammation and maintained for about 72 h.

### 2.2 Real‐Time PCR

We utilised the Trizol method to extract the total RNA from the hPDLFs. We read the RNA concentration extracted at the 260 nm position of the spectrophotometer. We transcribed the RNA into cDNA with a reverse transcription kit (Thermo, USA). Lastly, we put cDNA for reverse transcription polymerase chain reaction (RT‐PCR) with the Roche LightCycler480II real‐time PCR instrument (Applied Biosystems, Foster City, CA, USA) and SYBR Green Realtime PCR Master Mix (Kangwei, China).

### 2.3 Western Blot Analysis

On the 10% or 15% gel (Yase, China), 20 μg of the protein extract was electrophoresed in the lane. We transferred it to the polyvinylidene fluoride membrane. We blocked the membrane using 5% bovine serum albumin or 5% skim milk for one hour and then incubated overnight with MMP‐2, −1, and −3 antibodies at 4°C. Finally, we used the enhanced chemiluminescence system (Vazemy, China) and ImageJ software (National Institutes of Health) to detect the band intensity.

### 2.4 Enzyme‐Linked Immunosorbent Assay (ELISA)

To test the influence of different degrees of *P. gingivalis* ‐LPS on hPDLFs, we performed ELISA experiments with an ELISA kit (4A Biotech, Beijing, China). In the ELISA experiment, we got the MMP‐3, MMP‐1, and TIMP‐1 results. All steps were repeated 3 times for verification.

### 2.5 Wound Healing Assay

hPDLFs were plated into the 6‐well plates, maintained at 37°C for 6 h to allow hPDLFs to completely spread and adhere to the matrix. We drew one straight scratch to simulate the wound. HPDLFs were washed and used with the 1 mL growth medium to remove debris and smooth the scratch edges. And then we used the 5 mL medium dedicated to simulate an in vitro scratch test. We got images that used the phase contrast microscope to measure the hPDLFs cells wound place for initial and subsequent 6, 12, 24 h. Finally, we quantified all images using ImageJ software.

### 2.6 Cell Proliferation Assay

Used Cell Counting Kit‐8 (CCK‐8; Beyond, China) to measure cell proliferation. We seeded hPDLFs at a density of 3000 cells/well in the 96‐well plates. Then the cells were stimulated by *P. gingivalis* ‐LPS for 72 h. Finally, we added inhibitor pretreatment, 10 μL CCK‐8 per well. Plates were put in the humidified incubator for about 1.5 h, and absorbance at 450 nm was measured using the microplate reader.

### 2.7 Immunofluorescence

HPDLFs adhered to the round glass cover plate were fixed with the 4% buffered paraformaldehyde and permeabilised using the 0.1% Triton X‐100. HPDLFs cultivated with primary antibodies: Rabbit anti‐MMP‐2, −1, −3 (1:250; Abcam, Cambridge, UK), rabbit anti‐p38 (1:200; Abcam, Cambridge, UK). After cultivation with primary antibody, the dishes were washed, and secondary antibodies (Invitrogen, California, USA) were used to stain the cells. Washed dishes and scanned them on the Lycra fluorescence inverted microscope for watching. We used the ImageJ software to analyse results.

### 2.8 Statistical Analysis

Statistical analysis using GraphPad Prism 8.0 software, all data were expressed as mean ± SEM, and differences between groups were expressed using one‐way ANOVA, and the LSD test or Dunnett test was used for multiple groups according to homogeneity of variance. *p* < 0.05 is statistically significant.

## 3 Results

### 3.1 *P. gingivalis* ‐LPS Induces Expression of HPDLFs and MAPK P38‐Related Factors in Direct Proportionality

**FIGURE 1:**
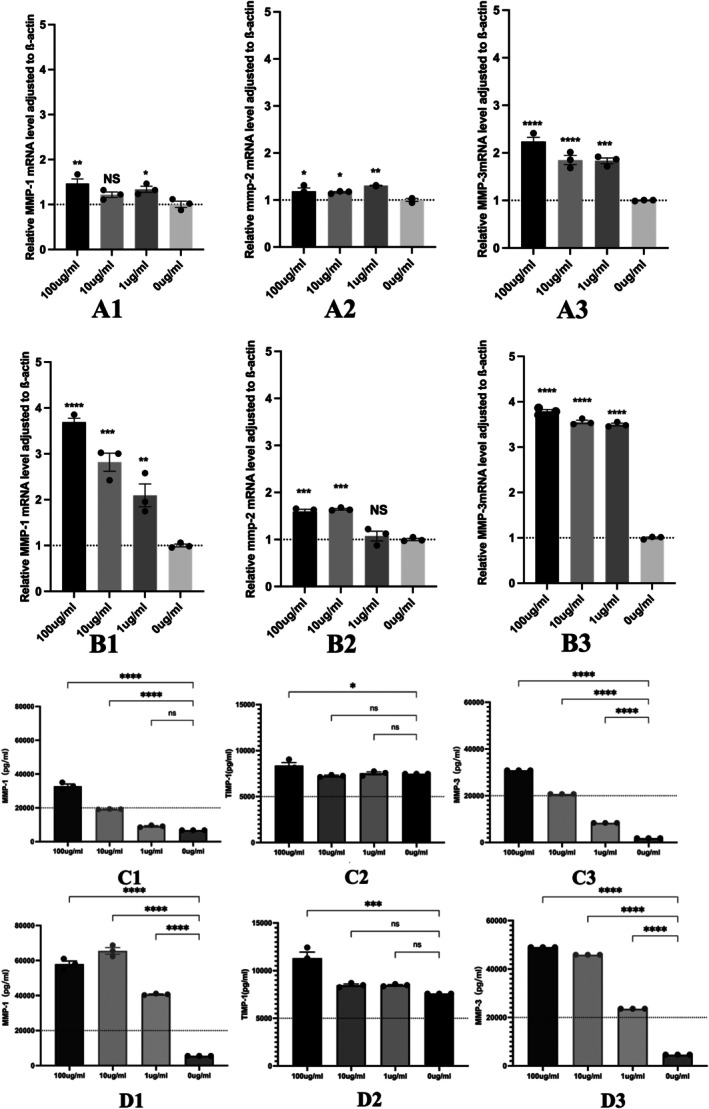
MMP‐1, −2, and −3 are increased in *P. gingivalis* ‐LPS‐induced hPDLF. (A1−A3) represent real‐time PCR analysis of mRNA expression of the MMP‐1, −2, and −3 at 24 h in hPDLFs treated with different concentrations of *P. gingivalis* ‐LPS, respectively. (B1−B3) represent real‐time PCR analysis of mRNA expression of the MMP‐1, −2, and −3 at 72 h in hPDLFs treated with different concentrations of *P. gingivalis* ‐LPS, respectively. (C1−C3) represent the ELISA analysis of MMP‐1, TIMP‐1, and MMP‐3 protein expression at 24‐h time points in hPDLFs tissues treated with different degrees of *P. gingivalis* ‐LPS. (D1−D3) represent ELISA analysis of MMP‐1, TIMP‐1, and MMP‐3 expression at 72‐h time points in hPDLFs tissues treated with the different degrees of *P. gingivalis* ‐LPS, respectively. Normal distributions were determined by ordinary one‐way ANOVA for each set of data, and then normal distributions were determined with the Shapiro–Wilk test. All Figs' Data are expressed in mean ± SEM (*n* = 3). *p < 0.05, **p < 0.01, ***p < 0.001. NS doesn't make any sense. NC, a group of cells that feed normally.

Our study explored whether hPDLF cells treated with different degrees of *P. gingivalis* ‐LPS would boost the expression of MMP. Our findings showed varied concentrations of the upregulation of downstream factors MMP‐2, −1, and −3. The third day, MMP‐2 in the 100ug/ml group and 10ug/ml group was more than 1.5 times, MMP‐3 in the 100ug/ml group and 10ug/ml group was nearly 4 times, and MMP‐1 in the 100ug/ml group and 10ug/ml group was nearly 4 times and 3 times (Figure 1A,B). Among them, the 100ug/ml group appeared highest expression.

This study was performed on human periodontal ligament fibroblasts (hPDLF) at 3 concentrations: 1, 10, and 100 μg/mL of the *P. gingivalis* ‐LPS. Cells were cultivated with the above degrees for 24 and 72 h. The control group used the equivalent volume of the complete culture medium. We collected cell culture supernatants after 24 and 72 h. We used the ELISA experiment to quantify MMP‐3, MMP‐1, and TIMP‐1 proteins.

The experiment showed that the hPDLFs cells cultivated with 1 μg/mL of the *P. gingivalis‐LPS* did not show any obvious changes in the TIMP‐1 and MMP‐1 within 24 h (*p* > 0.05). But an obvious increase in the MMP‐3 (*p* < 0.05). And 72 h later, MMP‐3 and MMP‐1 displayed an obvious upward trend (*p* < 0.05), whereas TIMP‐1 was basically unchanged (*p* > 0.05).

At a degree of 10 μg/mL, both at 24 and 72 h, MMP‐3 and MMP‐1 obviously improved (*p* < 0.05), while TIMP‐1 didn't show an obvious change (*p* > 0.05).

Cultivated with *P. gingivalis* LPS at 100 μg/mL, the expression of all measured proteins had obviously increased (*p* < 0.05) at 24 and 72 h. It indicated a dose‐dependent response (Figure 1C,D).

### 3.2 *P. gingivalis* ‐LPS Affects HPDLFs Characterisation via P38 MAPK

**FIGURE 2:**
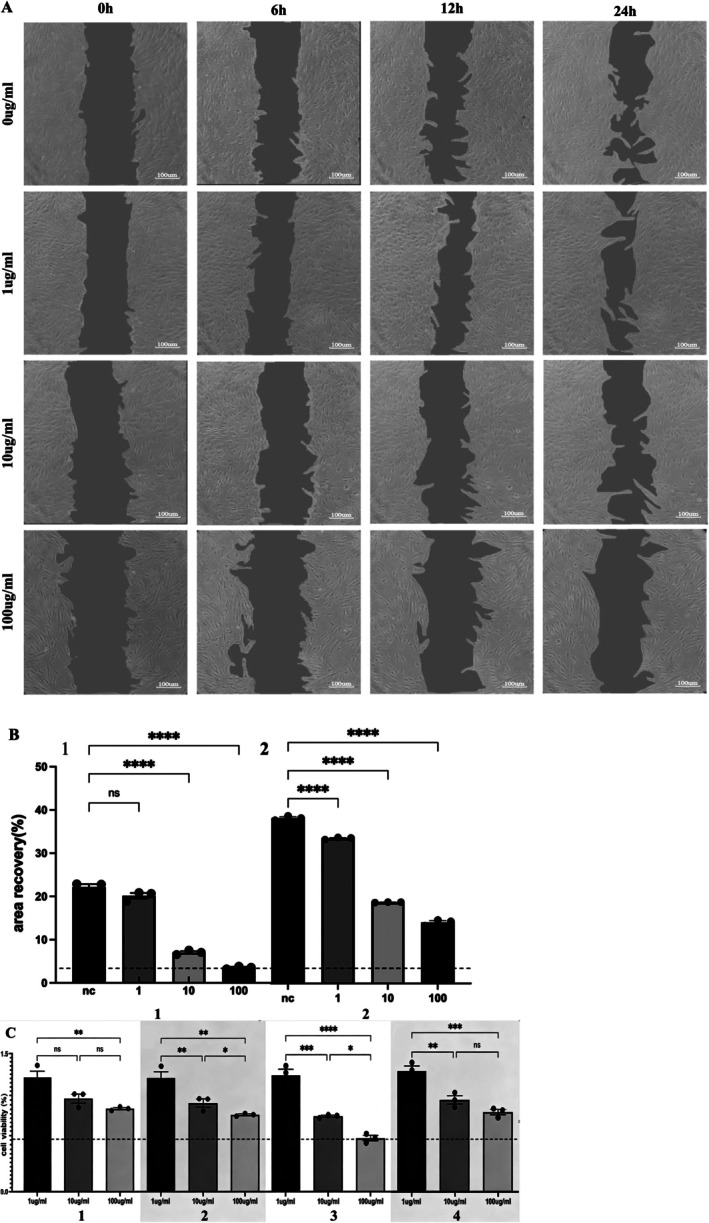
Induction of different degrees of the *P. gingivalis* ‐LPS inhibits hPDLFS migration and reproduction ability. (A, B) Cell scratch assay of hPDLs treated with different degrees of *P. gingivalis* ‐LPS (0, 1, 10, 100 μg/mL). Under the inverted microscope, use a shooting area with a scale bar = 50 μm. A is representative data, and B is quantitative. 1 is the 12‐h total data, showing the in vitro scratch test of different groups (*N* = 3), and 2 is the total data of 24 h, showing the in vitro scratch test of different groups (*N* = 3). (C) Effects of different degrees of the *P. gingivalis* ‐LPS (0, 1, 10, 100 μg/mL) on the survival rate of hPDLF. 1 is an experimental group of 24 h. 2 is a group of 48 h. 3 is a group of 72 h. 4 is a group of 96 h. Normal distributions were determined by ordinary one‐way ANOVA for each set of data, and then normal distributions were determined with the Shapiro–Wilk test.

Recently, studies elaborated on the influence of *P. gingivalis* ‐LPS on the hPDLFs’ functions, including proliferation and migration capabilities. To explore this, we conducted experiments to obtain the migration ability of the hPDLFs when handled with *P. gingivalis* ‐LPS. The outcomes showed a large reduction in the migration (Figure 2A,B). At the same time, experiments prompt the differences in the proliferation among the hPDLFs stimulated with different degrees of *P. gingivalis* ‐LPS (Figure 2C). hPDLF's proliferation will decrease progressively with increasing exposure time and *P. gingivalis* ‐LPS degrees.

### 3.3 Weaknesses of the MAPK Pathway Can Inhibit *P. gingivalis* LPS‐Induced Stimulation of P38 MAPK and Restore Function of the HPDLFs

**FIGURE 3:**
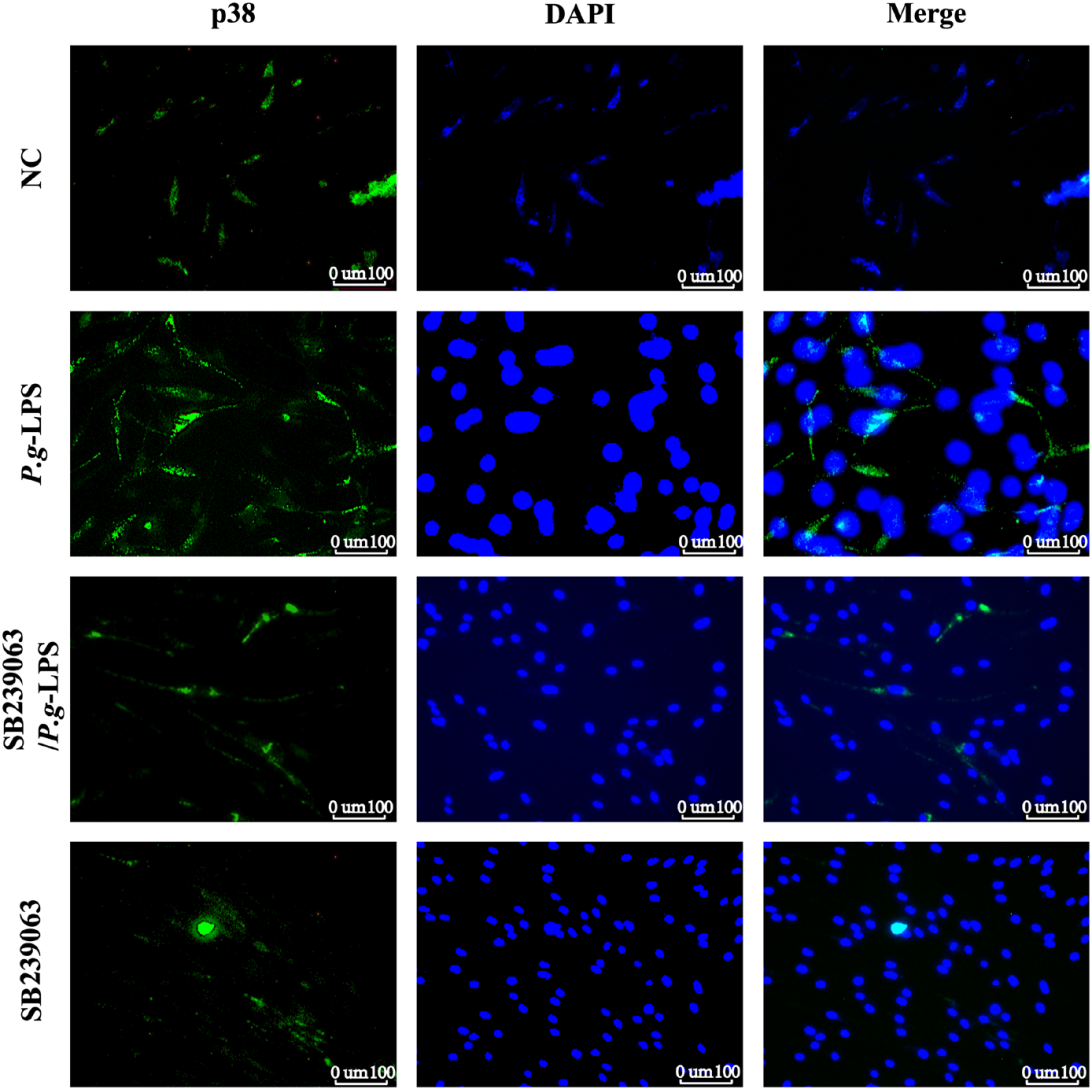
Inhibitors can inhibit the expression of p38 MAPK in hPDLFs. Experiment results showed that hPDLFs treated with inhibitors exhibited lower p38 MAPK expression than the control group. We conducted three independent fluorescence analyses to validate the result.

Founded on previous experiments, we chose to stimulate cells with the 100 μg/mL *P. gingivalis* ‐LPS for about 72 h. As part of the experimental group, additional pathway inhibition experiments were also conducted. The immunofluorescence experiments showed that the inhibitor SB239063 can inhibit the expression of p38 MAPK in hPDLFs (Figure 3).

### 3.4 P38 MAPK Inhibition Can Affect MMPs Protein Expression in HPDLFs Activated by P. Gingivalis‐LPS

**FIGURE 4:**
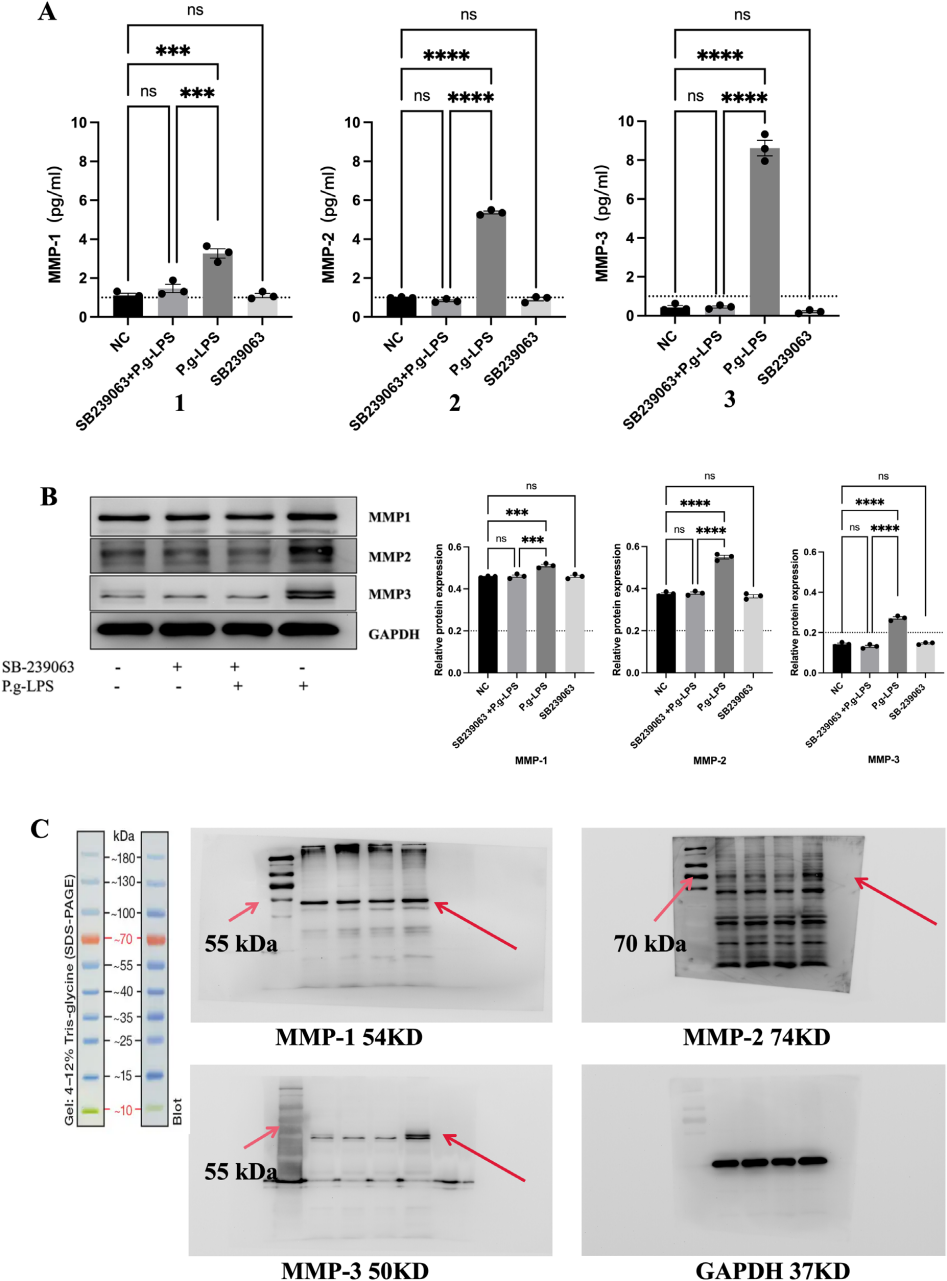
The effects of the p38 MAPK inhibitors and *P. gingivalis* ‐LPS on MMP‐1, −2, and −3 mRNA and protein expression in hPDLF. (A) Detection of mRNA in hPDLFs using RT‐PCR. *P. gingivalis*‐LPS stimulated gene expression of the MMP‐1, −2, and −3 mRNAs after 72 h of hPDLFs with or without p38 MAPK inhibitors. A1 stands for MMP‐1, A2 stands for MMP‐2, and A3 stands for MMP‐3. (B‐C) HPDLFs cultivated with *P. gingivalis* ‐LPS with or without inhibitor for about 72 h, and the MMP‐1, −2, and −3 proteins were measured by Western blot. The left side of the B layout is the representative data, the right side is the quantification, and C is the whole film diagram of the Western blot experiment. Normal distributions were determined by ordinary one‐way ANOVA for each set of data, and then normal distributions were determined with the Shapiro–Wilk test.

To clarify the relationship between MAPK and the expression and localisation of periodontitis‐related factors, we experimentally treated cells with p38 MAPK pathway inhibitors, and based on the above experimental results, we subsequently chose to treat them with P. gingivalis‐LPS 100 μg/mL for 72 h to assess the expression of downstream molecules. After inhibitor handling, a significant reduction in the relative mRNA expression of the individual MMPs appeared (Figure 4A).

In addition, we tested the expression of hPDLF‐related proteins in the presence of inhibitors to show the relationship between p38 MAPK and periodontitis. Experimental results showed that the expression of related matrix metalloproteinases was significantly reduced after blocking p38 MAPK (Figure 4B,C).

### 3.5 *P. Gingivalis* ‐LPS Affected Cell Characterisation by P38 MAPK Pathway

**FIGURE 5:**
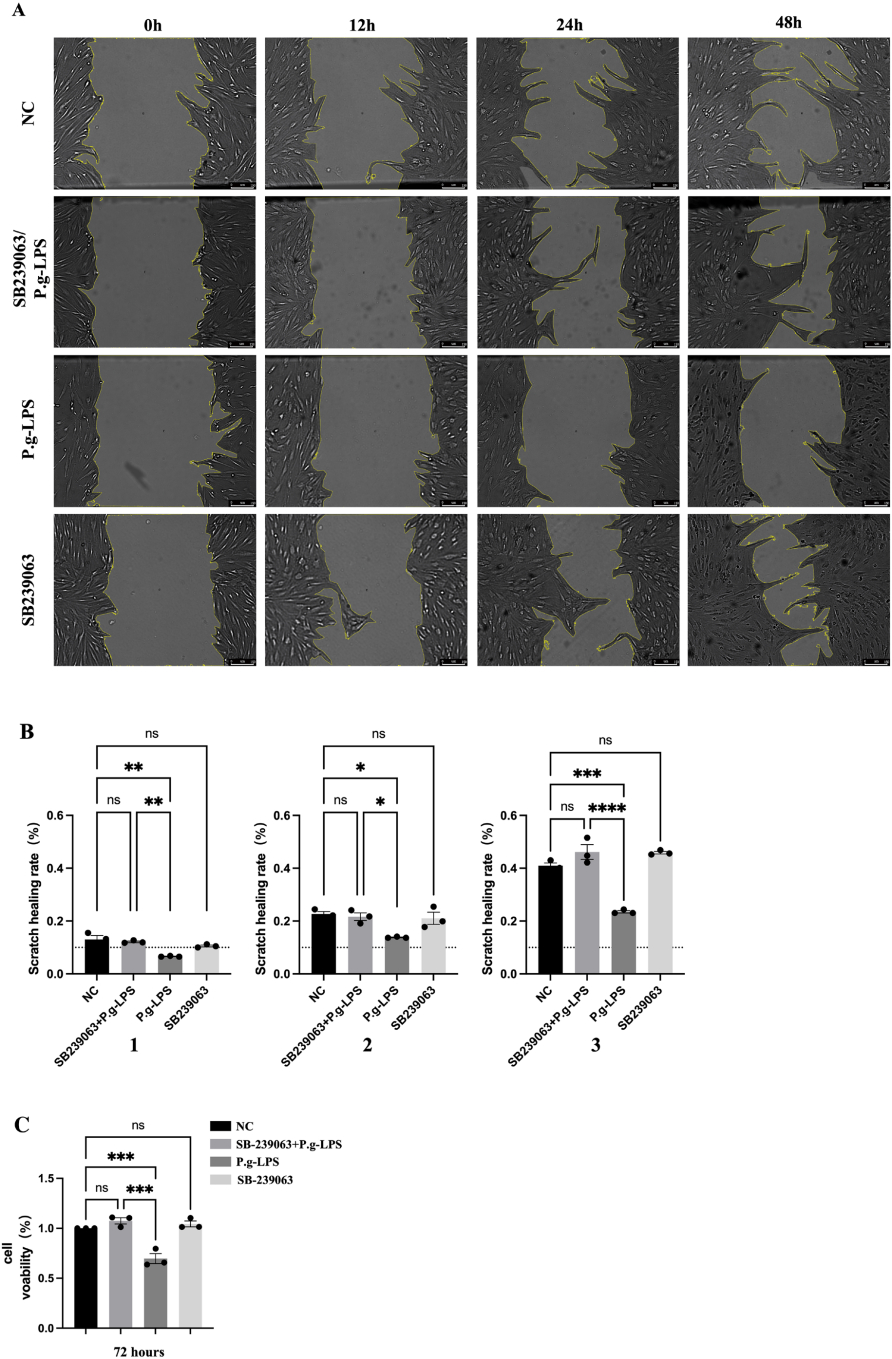
Migration and reproduction capacity of *P. gingivalis* ‐LPS and the p38 MAPK inhibitors on hPDLFs. (A, B) Wound scratch recovery with or without p38 MAPK inhibitors, divided into different groups at 12, 24, and 48 h, respectively, to simulate hPDLFs. Under the inverted microscope, use the shooting area, scale bar = 150 μm. A is representative data, and B is quantitative. 1 is the total data of 12 h, showing the in vitro scratch test of different groups (*N* = 3); 2 is the 24‐h total data, showing the in vitro scratch test (*N* = 3) in different groups. 3 is the total data at 48 h, showing the in vitro scratch test (*N* = 3) in different groups. (C) Effect of p38 MAPK inhibitor on hPDLFs survival. Normal distributions were determined by ordinary one‐way ANOVA for each set of data, and then normal distributions were determined with the Shapiro–Wilk test.

In the experiment, p38 MAPK pathway inhibitor (SB239063) was used for pretreatment to influence the P. gingivalis‐LPS on migration dysfunction and reduced proliferation ability of hPDLFs. The results showed that intervention with SB239063 improved cell proliferation and migration ability (Figure 5). Therefore, the results indicate that p38 MAPK participates in migration function and proliferation ability changes induced by P. gingivalis‐LPS.

## 4 Discussion

The gingival junction is the starting point of periodontitis and a battleground for the immune system to defend against the periodontal bacteria at the beginning and progression of the disease. MAPK cell signalling activates a variety of signalling cascades. p38 MAPK is a common upstream effector for many inflammatory cytokines, and recent studies have highlighted that the p38 MAPK signalling pathway is tightly related to periodontitis [11]. p38 MAPK can regulate cell apoptosis, differentiation, and autophagy [12], and its downstream targets include transcription factors, various kinases, and cytosolic proteins in the MAPK signalling cascade, which together regulate many important physiological/pathological effects. After phosphorylation, the p38 MAPK can activate multiple substrates in turn, affecting cell death, transcriptional regulation, cytokine secretion, and cytoskeletal remodelling [13]. The p38 family has a particularly significant impact on the development of chronic inflammation in periodontal tissues [14]. Activation of the p38 MAPK signalling directly or indirectly mediates inflammatory cytokine expression, which synergistically stimulates MMP production [15, 16]. This study aims to search for potential molecular targets after activation of the p38 MAPK pathway in hPDLFs, to help future studies better understand its impact on periodontal health. Similarly, little work has been done on the p38 MAPK pathway and MMP‐1, −2, −3 in the aetiology of periodontal disease.

This study induced inflammation in hPDLF by stimulation with different degrees of *P. gingivalis* ‐LPS. This method was selected based on the CellTiter 96 Aqueous assay and had no cytotoxic effects in cells treated with *P. gingivalis* ‐LPS. Investigations of hPDLF inflammation found significant changes compared to controls. Specifically, MMP‐1, −2, and −3 genes were expressed in *P. gingivalis* ‐LPS‐induced cells (see Figure 1A,B). In addition, MMP‐2, MMP‐1, and TIMP‐1 in hPDLFs cell culture supernatant were significantly increased, and their relative growth values were directly related to the concentration and induction time of *P. gingivalis* ‐LPS (see Figure 1C–E). In this study, the induction of the different concentrations of *P. gingivalis* ‐LPS on proliferation and movement of hPDLFs was investigated, and results have already verified that the *P. gingivalis* ‐LPS had different degrees of inhibitory effects on the proliferation and motility of hPDLFs, and with the increase of *P. gingivalis* ‐LPS concentration, proliferation and motility inhibition of hPDLFs were more obvious. This experiment highlights that MMP‐1, −2, and −3 are selectively regulated in inflammatory hPDLFs, and one of the vital factors in the ECM homeostasis is the matrix metalloproteinase, a key hydrolytic enzyme that affects periodontitis. Proteolytic enzymes can degrade a lot of cellular components and are closely related to the stability of the extracellular matrix [17]. Elevated MMP activity can cause extensive destruction of the basement membrane of the hPDLFs and the vascular endothelial cells, thereby enhancing periodontal ligament permeability, which can lead to periodontitis. MMP‐2 acts on the venous collagen in the extracellular matrix, affects intracellular collagen metabolism, and participates in the extracellular matrix inflammation. MMP‐1 degrades type 1 and type 3 collagen. MMP‐3 is involved in the periodontal matrix degradation [18]. In addition to alterations in gene and protein expression of MMP‐1, −2, and −3 mentioned above, MMP‐1, −2, and −3 also affect the activity of hPDLFs.

In addition, emerging literature suggests that the extracellular matrix changes in response to stress conditions. Under ordinary conditions, cells primarily use oxidative phosphorylation pathways to produce the energy needed for survival [19]. In inflammatory periodontal tissue, differences in how non‐immune and immune cells interact with bacteria and their components to produce inflammatory factors such as interleukins [20], and immune cells, e.g., T cells, neutrophils, and monocytes, are all activated by the pathogenic microorganisms, so cells switch from oxidative phosphorylation pathways to glycolysis to achieve rapid energy production to meet the increased need for matrix degradation [21], which is regulated by multiple signalling pathways and is crucial in bleeding cellular responses to the stress intensifying [22]. The inflammatory signalling pathway extends from the cell surface to the nucleus and is regulated by MAPK. Among the representative subfamilies of the MAPK pathway, p38 MAPK plays the key role. This pathway is essential for inflammation, infection stimuli, and signalling stress.

The p38 MAPK signalling pathway contributes significantly to extracellular matrix remodelling by regulating MMP‐1, −2, and −3 expression. We saw that the reduction and accumulation of MMP‐1, −2, and −3 may be related to the activity of the signalling pathway, and the expression of MMP‐2, −1, and −3 mRNAs in hPDLFs was significantly reduced after SB239063 effectively inhibited the p38 MAPK pathway. In addition, the effective inhibition of p38 MAPK led to a decrease in the MMP‐1, −2, and −3 proteins in *P. gingivalis* ‐LPS‐treated cells, and it was observed that the migration and proliferation of cells were restored after inhibition. Our findings suggest that extracellular matrix remodelling and degradation of *P. gingivalis‐LPS‐treated* hPDLFs is associated with the activation of p38 MAPK, and that cell migration and proliferation are also regulated by p38 MAPK. MMP‐1, −2, and −3 are key targets for inducing activation of the p38 MAPK pathway. Inflammation enhances the activation of p38 MAPK in hPDLFs in periodontitis, an important signalling pathway that regulates the expression of MMP‐1, −2, and −3 genes and proteins in cells, which are ultimately reflected in cell proliferation and motility. Therefore, the downregulation of MMP‐2, −1, and −3, and *P. gingivalis‐LPS* stimulation therapy has shown that biological functions are regulated from the transcription of the extracellular matrix to the protein level. Therefore, these data suggest that p38 MAPK regulates cellular inflammation in the extracellular matrix remodelling pathway, which may provide a new pathway for periodontal therapy. Furthermore, MAPK's role is not limited to modulating inflammatory responses; it also affects the activation of osteoclasts. Rapid phosphorylation of MAPK activates osteoclast function transcription factors, thereby regulating the gene expression of osteoclast differentiation [23], and hPDLFs select and attract the inflammatory cells, leading to their migration to the bone surface during the pathological process of periodontitis [24].

There is more and more evidence to prove that the role of MMP in regulating inflammation and immune responses is much more important than previouslyrecognised. At the site of periodontal inflammation, MMPs and MAPK pathways are involved in the cross‐ and self‐activation cascades and regulate the multiple inflammatory signalling molecules, but our current understanding of this process is far from complete. We will verify through more in vivo experiments in future.

## 5 Conclusions

Our findings reveal the significant effects of p38 MAPK and MMP‐1, −2, −3 on hPDLFs. These effects include activation of the intracellular MAPK signalling pathway, which regulates degradation of ECM and proliferation, invasion, and migration of hPDLFs through changes in MMP‐1, −2, and −3, and inhibiting release of pro‐inflammatory cytokines and MMPs through control of p38 MAPK, thereby restoring the remodelling of the extracellular matrix. Notably, p38 MAPK plays the key role in regulating inflammatory hPDLFs through the release of MMP‐1, −2, and −3, emphasising the potential role of the p38 MAPK in controlling periodontal disease by affecting cellular function, fibrosis, and collagenase activity. Activation of transcription factors that alter gene expression is essential for maintaining or restoring homeostasis to counteract environmental disturbances. p38 MAPK is important because it may participate in the host's immune defence response to periodontal disease‐associated microbial pathogens. Our approach exemplifies the unique goal of p38 MAPK in the regulation of periodontitis. By inhibiting p38 MAPK, thereby inhibiting MMP‐1, −2, and −3 expression, thereby reducing the degradation of the extracellular matrix, it may attenuate the abnormal inflammation that occurs in hPDLFs.

## Author Contributions

**Cheng Qian:** conceptualization, methodology, writing – original draft. **Yifan Gu:** data curation, investigation. **Shuyu Liu:** data curation, investigation. **Feiyan Gao:** data curation, investigation. **Zenan Xu:** data curation, investigation. **Rui He:** conceptualization, writing – review and editing. **Jintao Wang:** conceptualization, writing – original draft, writing – review and editing. **Jiahui Li:** data curation, investigation. **Liangjun Zhong:** writing – review and editing.

## Funding

This work was supported by the Biomedical Science and Technology Project of Hangzhou Science and Technology Bureau (2021WJCY287, 2021WJCY392) and the Key Project of Hangzhou Health Science and Technology Plan (ZD20210016).

## Disclosure

The authors have nothing to report.

## Ethics Statement

The authors have nothing to report.

## Consent

The authors have nothing to report.

## Conflicts of Interest

The authors declare no conflicts of interest.

## Data Availability

The data that support the findings of this study are available from the corresponding author upon reasonable request.
